# Supplementary material for: The Connectional Diaschisis and Normalization of Cortical Language Network Dynamics After Basal Ganglia and Thalamus Stroke
Source: Neurobiol Lang (Camb). 2025 Sep 17;6:NOL.a.16. doi: 10.1162/NOL.a.16 (PMC12459977; doi:10.1162/NOL.a.16)
Supplement: Supplementary file 1 [file nol-6-1-16-s001.pdf]

The connectional diaschisis and normalization of cortical language network dynamics after basal ganglia and thalamus stroke

Supplementary Table 1

Supplementary Table 1 Anatomical regions， language-related behavioral domains, and paradigm classes of the language network. Coordinates are in the standard Montreal Neurologic Institute Space.

ID	*x*	*y*	*z*	**anatomy**	**Behavioral domains**	**Paradigm classes**
1	-4.6	15.7	53.2	SFG	Execution. Speech, Phonology, Semantics, Speech	Word Generation (Covert and Overt)
2	7.0	16.4	54.7	SFG	Homolog of node 1	-
17	-41.9	13.6	36.4	MFG	Phonology, Semantics	Semantic. Monitor/Discrimination, Word Generation (Covert)
18	42.2	11.9	38.4	MFG	Homolog of node 17	-
29	-45.6	13.0	23.4	IFG	Phonology, Semantics, Speech, and Syntax	Phonological. Discrimination, Semantic. Monitor/Discrimination
30	45.0	15.7	25.0	IFG	Homolog of node 29	-
31	-47.6	31.6	13.6	IFG	Phonology, Semantics, Speech, and Syntax	Phonological. Discrimination, Semantic. Monitor/Discrimination, Word Generation (Covert and Overt)
32	47.8	35.1	13.2	IFG	Homolog of node 31	-
33	-52.2	22.5	11.4	IFG	Semantics, Speech, and Syntax	Reading (Covert), Semantic. Monitor/Discrimination, Word Generation (Covert and Overt)
34	54.2	23.9	11.8	IFG	Homolog of node 33	-
35	-49.1	36.3	-2.8	IFG	Semantics, Speech, and	Semantic. Monitor/Discrimination,

					Syntax	Word Generation (Covert)
36	50.8	36.8	-0.7	IFG	Homolog of node 35	-
37	-39.5	22.9	3.7	IFG	Phonology, Semantics, Speech, and Syntax	Semantic. Monitor/Discrimination, Word Generation (Covert)
38	42.1	22.0	3.2	IFG	Homolog of node 37	-
39	-51.3	13.2	6.0	IFG	Phonology, Semantics, Speech	Music. Comprehension/Production, Recitation/Repetition. (Covert), Word Generation (Covert)
40	53.6	14.3	11.8	IFG	Homolog of node 39	-
53	-49.5	-7.1	38.8	PrG	Execution. Speech	Reading (Overt), Recitation/Repetition. (Overt)
54	54.9	-2.0	33.3	PrG	Execution. Speech	Reading (Overt), Recitation/Repetition. (Overt)
63	-49.1	4.7	30.5	PrG	Orthography, Phonology, Semantics, Speech and Syntax	Phonological. Discrimination, Reading (Covert)
64	51.1	7.2	30.9	PrG	Homolog of node 63	-
71	-53.7	-32.1	12.4	STG	Execution. Speech, Phonology, and Speech	Music. Comprehension/Production, Passive Listening, Phonological. Discrimination, Reading (Overt), Recitation/Repetition. (Covert and Overt)
72	54.5	-23.7	10.6	STG	Execution. Speech, Phonology	Music. Comprehension/Production, Passive Listening, Phonological. Discrimination, Recitation/Repetition. (Overt)
73	-50.1	-10.3	1.1	STG	Execution. Speech, Phonology, Speech	Music. Comprehension/Production, Passive Listening, Phonological. Discrimination, Reading (Overt), Recitation/Repetition. (Overt)

74	51.1	-3.7	-0.9	STG	Execution. Speech,	Music. Comprehension/Production, Passive Listening, Recitation/Repetition. (Overt)
75	-62.8	-32.8	7.4	STG	Execution. Speech, Phonology, Semantics, Speech	Passive Listening, Phonological. Discrimination, Reading (Overt), Semantic. Monitor/Discrimination
76	66.5	-20.8	6.6	STG	Execution. Speech, Phonology, Speech	Music. Comprehension/Production, Passive Listening, Phonological. Discrimination, Reading (Overt)
77	-45.1	10.5	-19.4	STG	Homolog of node 78	-
78	47.1	12.3	-19.7	STG	Speech	Film Viewing, Passive Listening
79	-55.1	-3.2	-10.1	STG	Execution. Speech, Phonology, Semantics, Speech	Music. Comprehension/Production, Passive Listening, Phonological. Discrimination, Reading (Overt)
80	55.8	-12.5	-5.2	STG	Execution. Speech, Phonology, Semantics, Speech	Music. Comprehension/Production, Passive Listening, Phonological. Discrimination, Reading (Overt), Semantic. Monitor/Discrimination
81	-65.2	-30.9	-11.3	MTG	Semantics	Semantic. Monitor/Discrimination
82	64.5	-29.2	-13.2	MTG	Homolog of node 81	-
83	-53.2	2.2	-29.6	MTG	Language	Semantic. Monitor/Discrimination, Reading (Covert)
84	51.1	5.7	-31.8	MTG	Language	Passive Listening
85	-58.9	-57.6	4.3	MTG	Semantics and Syntax	Semantic. Monitor/Discrimination, Word Generation (Overt)
86	60.1	-53.3	2.9	MTG	Homologues of node 85	Film Viewing
87	-58.5	-19.8	-9.4	MTG	Phonology, Semantics, Speech, and Syntax	Passive Listening, Phonological. Discrimination, Reading (Covert), Semantic. Monitor/Discrimination

88	58.3	-15.4	-10.1	MTG	Semantics and Speech	Passive Listening, Phonological. Discrimination
89	-45.5	-26.7	-26.1	ITG	Orthography and Semantics	Reading (Covert), Semantic. Monitor/Discrimination
90	45.8	-14.6	-32.4	ITG	Homolog of node 89	-
91	-50.5	-57.0	-14.1	ITG	Phonology and Semantics	Naming (Overt)
92	53.5	-52.4	-18.5	ITG	Phonology and Semantics	-
93	-43.7	-2.9	-41.4	ITG	Semantics	Semantic. Monitor/Discrimination
94	40.5	-2.9	-41.4	ITG	Homolog of node 93	-
97	-55.2	-60.3	-6.0	ITG	Semantics and Speech	Film Viewing, Naming (Overt)
98	54.2	-56.9	-8.6	ITG	Homolog of node 97	-
99	-58.8	-42.1	-16.0	ITG	Orthography and Semantics	Naming (Overt), Reading (Covert), Semantic. Monitor/Discrimination, Word Generation (Overt)
100	60.5	-40.5	-17.1	ITG	Homolog of node 99	-
101	-54.9	-30.3	-27.4	ITG	Semantics	-
102	53.7	-30.3	-26.3	ITG	Homolog of node 101	-
103	-32.4	-16.6	-32.3	FuG	Semantics and Speech	Naming (Overt), Semantic. Monitor/Discrimination
104	33.1	-14.6	-34.1	FuG	Semantics	Naming (Overt), Semantic. Monitor/Discrimination
105	-30.6	-64.4	-14.1	FuG	Orthography, Semantics, Speech	Naming (Covert and Overt)
106	31.3	-61.4	-13.7	FuG	Language	Naming (Covert and Overt)
107	-42.3	-50.9	-17.3	FuG	Orthography, Phonology, Semantics, Speech	Naming (Covert and Overt), Phonological. Discrimination, Reading (Covert) and Semantic. Monitor/Discrimination
108	42.7	-49.1	-18.6	FuG	Orthography, Semantics	Naming (Covert)

113	-28.3	-32.6	-16.9	PhG	Semantics	Naming (Overt), Semantic. Monitor/Discrimination
114	28.8	-30.7	-17.5	PhG	Homolog of node 113	Passive Listening, Semantic. Monitor/Discrimination
121	-54.4	-39.8	4.2	pSTS	Phonology, Semantics, Speech, Syntax	Passive Listening, Phonological. Discrimination, Reading (Covert), Semantic. Monitor/Discrimination, Word Generation (Covert and Overt)
122	52.9	-36.8	3.1	pSTS	Execution. Speech, Phonology, Semantics, and Speech	Passive Listening, Phonological. Discrimination, Reading (Overt)
123	-52.4	-50.3	10.8	pSTS	Orthography, Semantics, Speech, and Syntax	Reading (Covert), Semantic. Monitor/Discrimination
124	56.5	-40.1	12.5	pSTS	Homologues of node 123	Passive Listening
143	-46.8	-64.7	25.8	IPL	Language	Semantic. Monitor/Discrimination,
144	53.0	-54.1	24.4	IPL	Homolog of node 143	-
155	-50.4	-15.8	42.1	PoG	Execution. Speech	Recitation/Repetition. (Overt)
156	50.3	-14.2	43.7	PoG	Execution. Speech	Reading (Overt), Recitation/Repetition. (Overt)
157	-55.8	-14.0	16.2	PoG	Execution. Speech	Recitation/Repetition. (Overt)
158	55.2	-10.2	15.0	PoG	Execution. Speech	Recitation/Repetition. (Overt)

SFG, superior frontal gurus; *MFG*, middle frontal gyrus; *IFG*, inferior frontal gyrus; *OrG*, orbital gyrus; *PrG*, precentral gyrus; *STG*, superior temporal gyrus; *MTG*, middle temporal gyrus; *ITG*, inferior temporal gyrus; *FuG*, fusiform gyrus; *PhG*, hippocampal gyrus; *pSTS*, posterior superior temporal sulcus; *IPL*, inferior parietal lobule. *PoG*, postcentral gyrus. The ID is the number of the parcel in the Brainnetome atlas (Fan et al., 2016). Here we only summarized the language-related behavioral domains and paradigm classes; the full behavioral domains and paradigm classes for each node are available at http://atlas.brainnetome.org/bnatlas.php.

Supplementary Figures

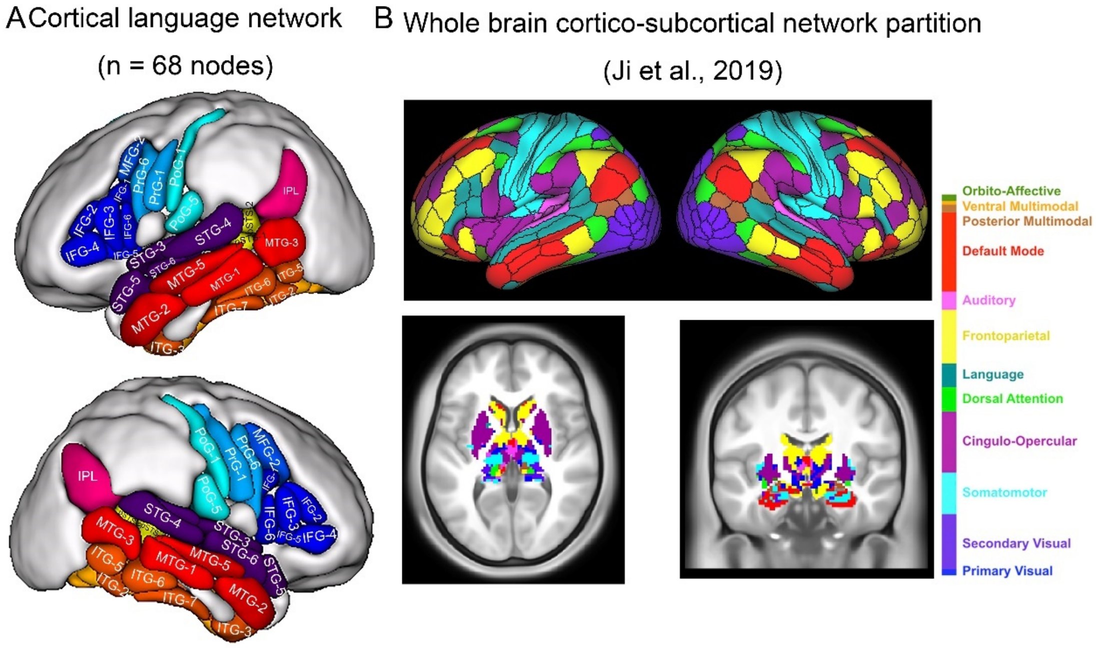

Supplementary Figure 1. The cortical language network and the 11 large-scale brain networks. A: 68 cortical regions involved in language processing were selected from the Brainnetome atlas (Fan et al., 2016). B: To delineate the resting-state brain networks encompassing the cortical language network we defined, we have redrawn the 12 whole-brain resting-state networks based on functional connectivity as defined by Ji et al. (2019). It can be observed that the language network we defined includes the typical language network (IFG and STG), auditory network, the default mode network, and the ventral part of the somatomotor network. These networks each have corresponding regions in the subcortical areas.

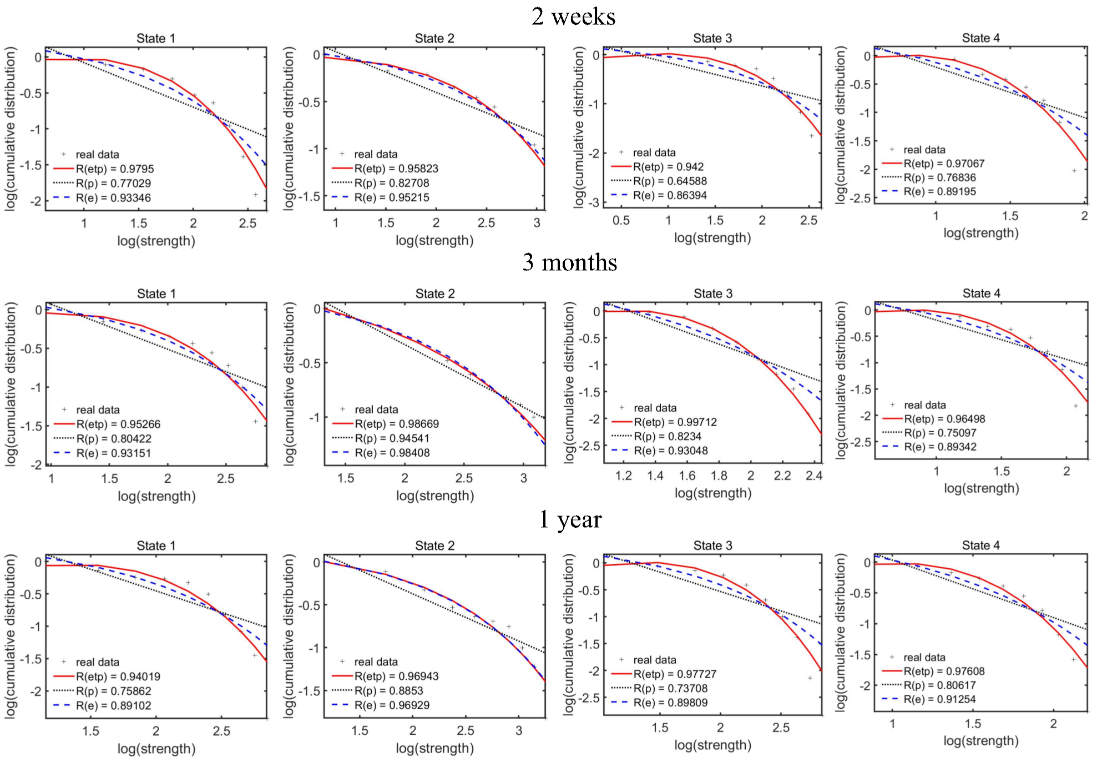

Supplementary Figure 2. The log-log plots of the cumulative nodal strength distributions. The plus sign (black) represents observed data, the solid line (red) is the fit of the exponentially truncated power-law, $P(x) \sim x^{\alpha-1}\exp\left({x}/{x_c}\right)$, the dashed line (blue) is an exponential, $P(x) \sim \exp\left({x}/{x_c}\right)$, and the dotted line (black) is a power-law, $P(x) \sim x^{\alpha-1}$. R^2 was calculated to assess the goodness-of-fit. A larger value indicates a better fitting: R_{etp}, R^2 for the exponentially truncated power-law; R_e, R^2 for the exponential; R_p, R^2 for the power-law fit. The exponentially truncated power-law is the best fitting for all 4 states, which suggest a long-tailed broad-scale topologies and a large proportion of network connectivity will be concentrated on a subset node (i.e., hubs).

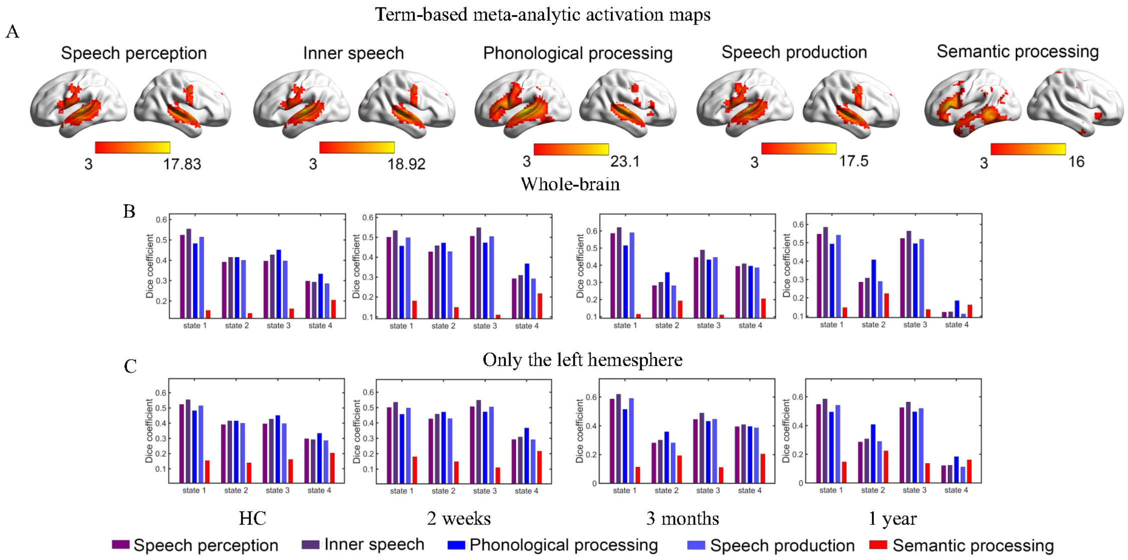

Supplementary Figure 3. Functional relevance of hub distributions. A. The meta results of speech perception, inner speech, phonological processing, speech production, and semantic processing were from 'NeuroQuery' (https://neuroquery.org/) (Dockes et al., 2020). Each map was thresholded at Z = 3 (a typical value used by NeuroQuery) for illustrative purposes and only positive results were shown. B&C: the dice coefficients between binary images of hub nodes and meta results. Considering the left-lateralized activations of meta results, the dice coefficients were calculated at the whole-brain level and in the left hemisphere.

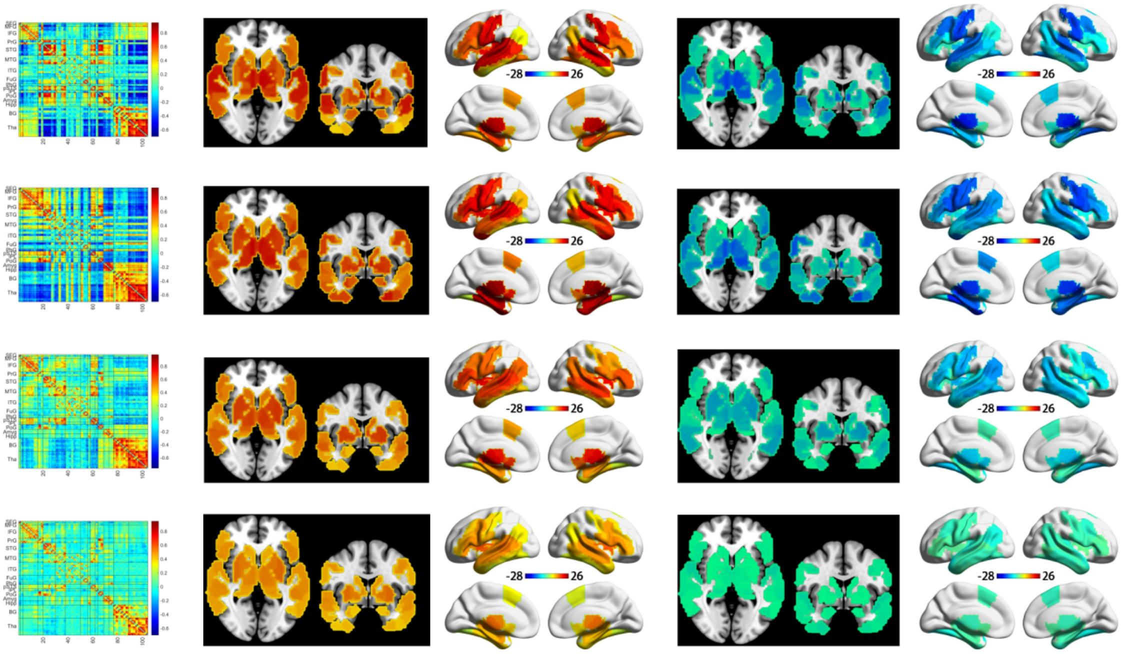

Supplementary Figure 4. The dynamic functional connectivity between cortical language areas and subcortical areas of HCs (n = 25). Figures in the left column show the four temporal-reoccurring DFC states. Figures in the right four columns show the nodal strengths of positive and negative connections in volumetric and surface space.

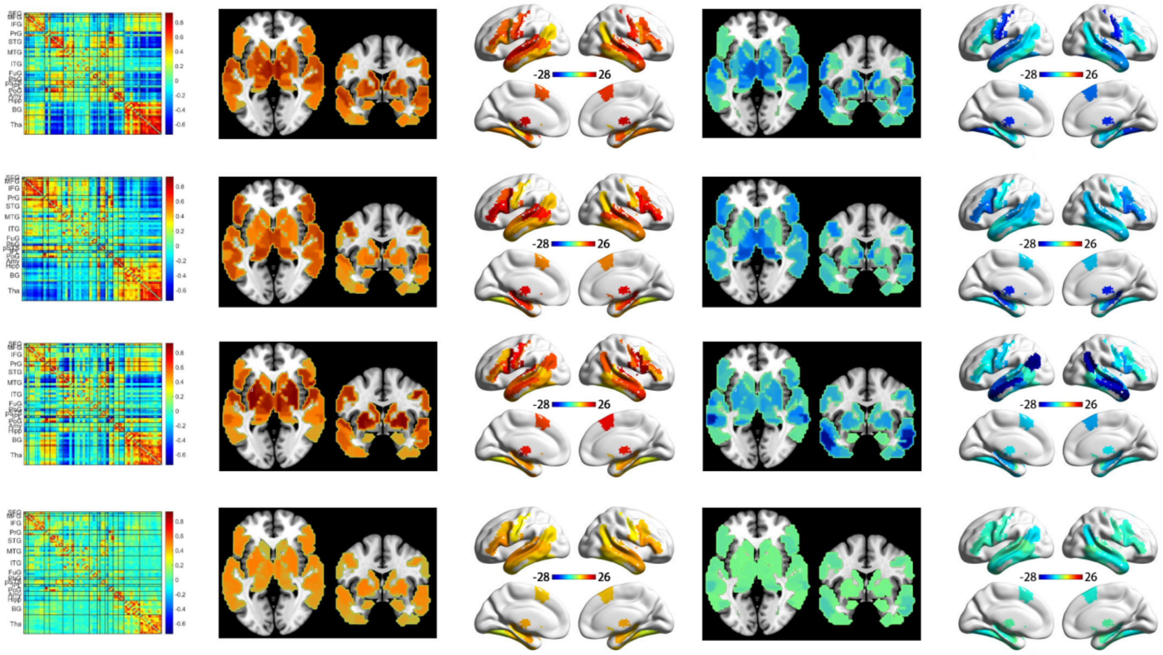

Supplementary Figure 5. The cortico-subcortical dynamics in young healthy subjects (n = 192).

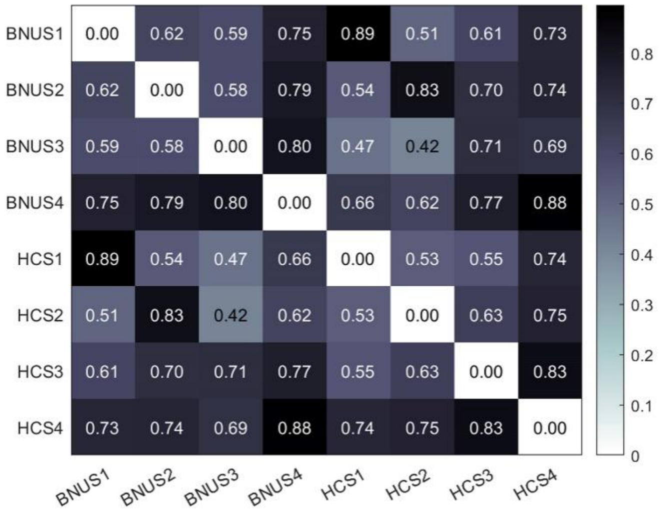

Supplementary Figure 6. The spatial correlation of the four states of cortico-subcortical dynamics in HCs (n = 25) and young healthy subjects (n = 192).

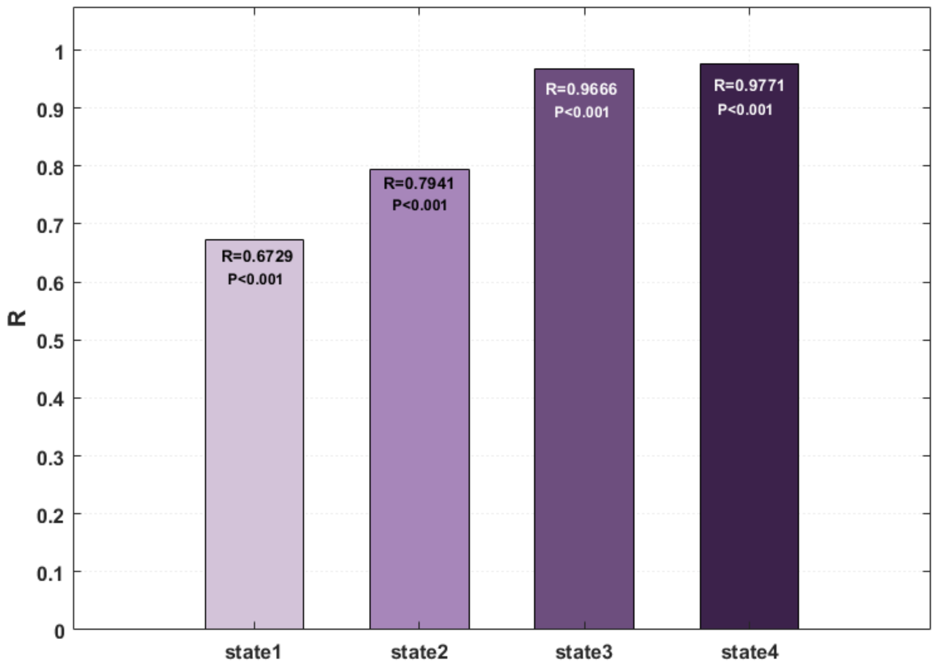

Supplementary Figure 7. The spatial correlation between the four states of cortical language networks and the cortical components of the four states of cortico-subcortical network.

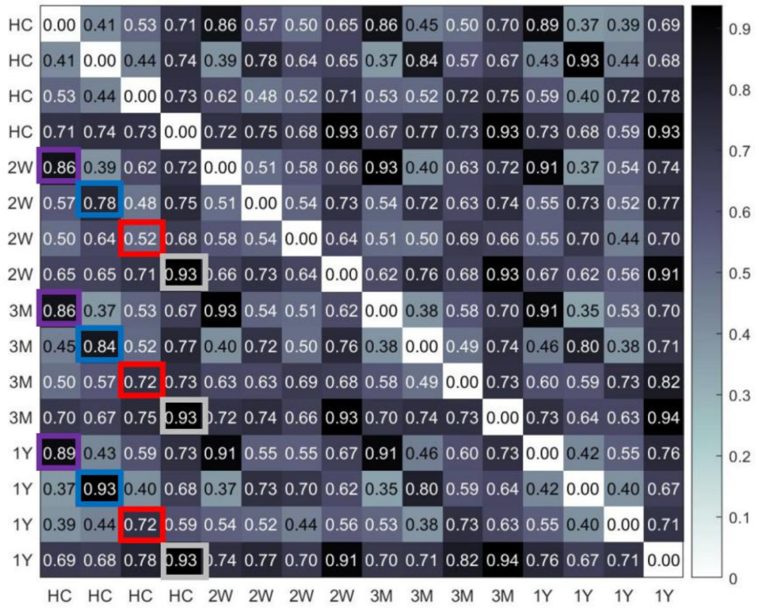

Supplementary Figure 8. The spatial correlation coefficients among the four states of HCs and patients. During language recovery, an increase in spatial similarity between patients and HCs was observed, especially for states 2 and 3.

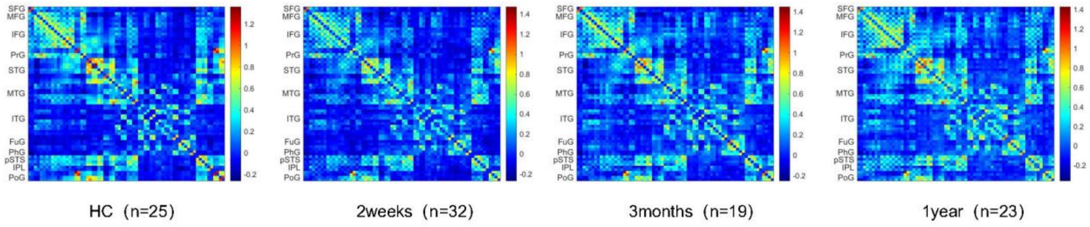

Supplementary Figure 9. The mean static functional connectivity of HCs and patients after stroke.
